# ICE COLD ERIC – International collaborative effort on chronic obstructive lung disease: exacerbation risk index cohorts – Study protocol for an international COPD cohort study

**DOI:** 10.1186/1471-2466-9-15

**Published:** 2009-05-06

**Authors:** Lara Siebeling, Gerben ter Riet, Willem M van der Wal, Ronald B Geskus, Marco Zoller, Patrick Muggensturm, Irena Joleska, Milo A Puhan

**Affiliations:** 1 Department of General Practice, Academic Medical Centre, University of Amsterdam, Amsterdam, The Netherlands; 2 Department of Clinical Epidemiology, Biostatistics and Bioinformatics, Academic Medical Centre, University of Amsterdam, Amsterdam, The Netherlands; 3 Department of General Practice, University of Zurich, Zurich, Switzerland; 4 Horten Centre for patient-oriented research, University of Zurich, Zurich, Switzerland; 5 Department of Epidemiology, Bloomberg School of Public Health, Johns Hopkins University, Baltimore, MD, USA

## Abstract

**Background:**

Chronic Obstructive Pulmonary Disease (COPD) is a systemic disease; morbidity and mortality due to COPD are on the increase, and it has great impact on patients' lives. Most COPD patients are managed by general practitioners (GP). Too often, GPs base their initial assessment of patient's disease severity mainly on lung function. However, lung function correlates poorly with COPD-specific health-related quality of life and exacerbation frequency. A validated COPD disease risk index that better represents the clinical manifestations of COPD and is feasible in primary care seems to be useful. The objective of this study is to develop and validate a practical COPD disease risk index that predicts the clinical course of COPD in primary care patients with GOLD stages 2–4.

**Methods/Design:**

We will conduct 2 linked prospective cohort studies with COPD patients from GPs in Switzerland and the Netherlands. We will perform a baseline assessment including detailed patient history, questionnaires, lung function, history of exacerbations, measurement of exercise capacity and blood sampling. During the follow-up of at least 2 years, we will update the patients' profile by registering exacerbations, health-related quality of life and any changes in the use of medication. The primary outcome will be health-related quality of life. Secondary outcomes will be exacerbation frequency and mortality. Using multivariable regression analysis, we will identify the best combination of variables predicting these outcomes over one and two years and, depending on funding, even more years.

**Discussion:**

Despite the diversity of clinical manifestations and available treatments, assessment and management today do not reflect the multifaceted character of the disease. This is in contrast to preventive cardiology where, nowadays, the treatment in primary care is based on patient-specific and fairly refined cardiovascular risk profile corresponding to differences in prognosis. After completion of this study, we will have a practical COPD-disease risk index that predicts the clinical course of COPD in primary care patients with GOLD stages 2–4. In a second step we will incorporate evidence-based treatment effects into this model, such that the instrument may guide physicians in selecting treatment based on the individual patients' prognosis.

**Trial registration:**

ClinicalTrials.gov Archive NCT00706602

## Background

The majority of Chronic Obstructive Pulmonary Disease (COPD) patients are treated in primary care [1]. COPD is a leading cause of morbidity and mortality worldwide with an increasing prevalence, especially in women and an increasing economic and social burden. Management of COPD is challenging because of the diversity of clinical manifestations and available treatments [2]. General practitioners (GP) need to select those treatments that will improve the individual patient's health status and clinical course (prognosis) most. However, recent surveys on COPD management show that a majority of physicians do not consider the patient's severity of disease when prescribing COPD treatments and that current medical practice deviates substantially from the GOLD guidelines [3-6]. For example, most patients receive inhaled steroids although only patients with frequent exacerbations and/or Global Initiative for Chronic Obstructive Lung Disease (GOLD) stage 3 and 4 may benefit [7-9]. Conversely, pulmonary rehabilitation and long-term oxygen are prescribed rarely for patients with advanced COPD despite strong evidence supporting their effectiveness [3,4,10,11]. These results suggest that GPs may not have methods available to sufficiently consider severity of disease and projected prognosis given particular treatment decisions.

Assessment of disease severity should not focus solely on lung function because it correlates, at best, moderately with clinically relevant outcomes such as health-related quality of life, dyspnea, or exacerbations. An increasing body of evidence shows that additional information should be ascertained that better reflects disease severity [12-15]. And indeed, in their latest guidelines, the European Respiratory Society and American Thoracic Society state that "it is accepted that a single measurement of Forced Expiratory Volume in one second (FEV1) incompletely represents the complex clinical consequences of COPD" and that additional parameters should be ascertained [2].

There is one index, the BODE index, that follows this line by incorporating FEV_1_, dyspnea, body mass index and exercise capacity (6-minute walking test) into a single index [14]. It provides information about COPD patients' prognosis and may help tailoring therapy to a particular patient's risk. However, although the BODE index (B = Body Mass Index, O = Obstruction, D = Dyspnea and E = Exercise capacity) represents a substantial advance in COPD evaluation, it has important limitations hindering its application in primary care. First, the 6-minute walking test is currently not easily applicable outside a rehabilitation setting. Second, the development process of the BODE index was not optimal; potentially important information about disease severity such as history of exacerbations, current smoking or arterial oxygen pressure was not considered in the development process. Third, the BODE index predicts mortality, which hardly guides physicians in selecting treatments. Fourth, the BODE index was developed in COPD clinics and it has not been validated in any clinical or primary care population. Often, risk indices perform worse in other populations or settings, which could lead to inadequate predictions followed by suboptimal decisions affecting patient management and possibly outcome [16]. These limitations currently make the BODE index unsuitable for primary care settings. A recent survey illustrates this: only 28% of GPs said they had heard about the BODE index and 0% knew any of its components [5].

Therefore, a disease risk index for COPD patients should be developed using more advanced methods and one that is applicable in primary care. The European Respiratory Society and American Thoracic Society also call for a multivariable disease risk tool; "a staging system that could offer a composite picture of disease severity is highly desirable, although it is currently unavailable" [2]. This is also in line with the growing interest in the medical community for risk-stratification based on multivariable evaluation.

**Figure 1:**
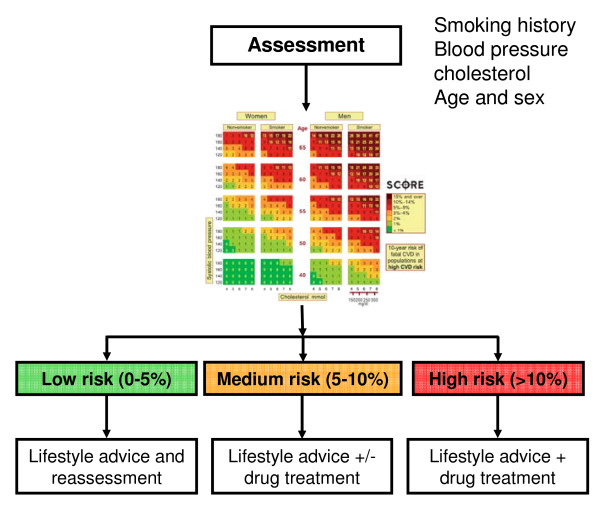
**Cardiovascular (CV) risk assessment and treatment choice**; based on several parameters, patients fall into a specific colored box (details are illegible) and the color corresponds to a risk profile (green = low risk, orange = medium risk and red = high risk). According to that risk profile, treatment recommendation(s) are given.

**Figure 2:**
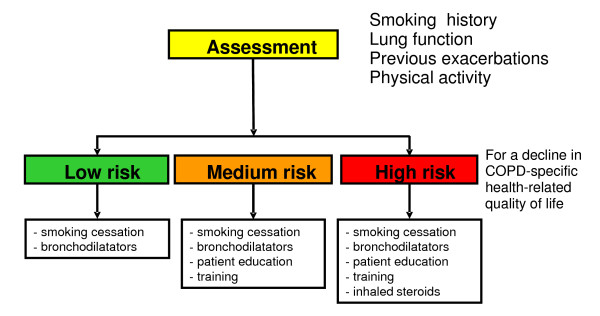
**COPD exacerbation risk assessment and treatment choice (example)**; based on several parameters, patients fall into a specific risk profile (green = low risk, orange = medium risk and red = high risk). According to that risk profile, treatment advice(s) are given.

Respiratory medicine might gain from emulating the approach taken in preventive cardiology. Cardiovascular risk can be estimated based on several parameters, as shown in Figure 1, which in turn guide selection of treatments (so-called risk index-guided treatment). Correspondingly, future COPD management might look as shown in Figure 2. Assessment by a risk index would inform the optimal selection of COPD treatments targeting current symptoms and limitations as well as slowing down disease progression. Thus, COPD management would be individualized and, thereby, on average, tailored better to a particular patient's needs.

In conclusion, the aim of the ICECOLD ERIC study is to develop and validate a practical COPD disease risk index that predicts the clinical course of COPD in primary care settings. In this study, we will identify important risk predictors for health-related quality of life, exacerbation risk and mortality. In a second step we will try to incorporate treatment effects into the model using data from the study itself and (external) data from pertinent RCTs and meta-analyses. This instrument will guide treatment on the individual patient's COPD severity.

## Methods/Design

### Study design

**Figure 3:**
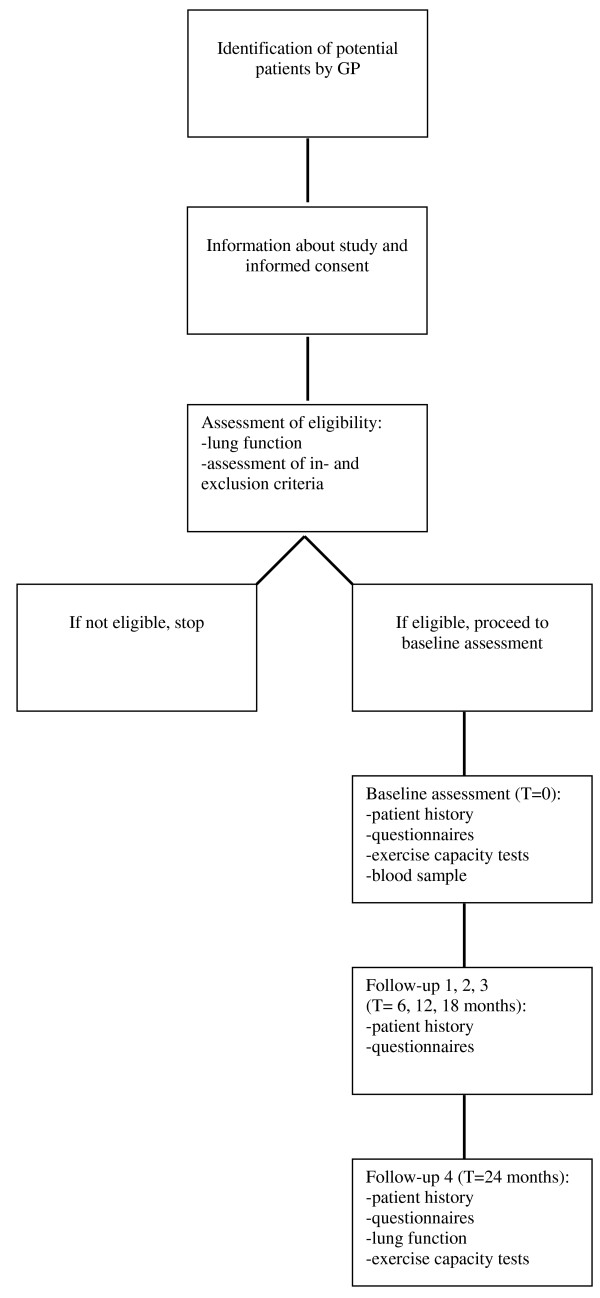
**flow chart**.

We will conduct two prospective cohort studies (Swiss and Dutch counterparts), following a similar study protocol. We hope to be able to collect data from other cohort studies with similar protocols at a later stage. Figure 3 contains a flow chart of the study. The study has been approved of by all local ethic committees and is registered on the website  with the identifier: NCT00706602.

### Outcomes

The primary outcome, for which the risk index will be developed, will be COPD-specific health-related quality of life as measured by the Chronic Respiratory Questionnaire (CRQ). COPD-specific health-related quality of life is one of the main outcomes used in prognostic COPD research whether observational or interventional [11-13]. Secondary outcomes will include COPD exacerbations requiring medical treatment and mortality.

### Population

We will include patients ≥ 40 years of age with COPD in GOLD stage 2–4 (postbronchodilator FEV1/FVC ≤ 0.70 and postbronchodilator FEV1 ≤ 80% of predicted), who are able to complete the baseline assessment and who have been free of exacerbation for at least four weeks. Exclusion criteria will be a life expectancy of ≤ 12 months (predicted by the patient's GP), dementia, psychosis or other psychiatric morbidity that invalidates assessment of patient-reported parameters such as health-related quality of life, physical activity or dyspnea, and inability to complete the baseline assessment due to language difficulties. These criteria will be evaluated in cooperation with the participating GPs by browsing the patients' charts and during the eligibility testing.

### Recruitment of GPs and patients

In Switzerland, we will invite GPs from private practice in the cantons of Zurich and St. Gallen using a two-stage strategy. In a first step, we will involve GPs from the North-Eastern part of Switzerland whom we know to have an active interest in research activities. Through these GPs we will identify more GPs potentially willing to participate (snowballing approach). In a second step, we will invite the latter to participate. We will send them a brochure describing the background and aim of the study as well as what participation entails. Also, we will outline the benefits of participation. Participating GPs will identify potentially eligible patients through electronic or paper-based patient charts. For electronic patient charts, GPs will search with the key terms "copd", "chronic bronchitis", "emphysema", "asthma" and a combination of "smoking", "≥ 40 years of age" and "male" to identify patients with obstructive airway disease. Paper-based patient charts will be screened by hand. The participating GPs inform potentially eligible patients about the purpose of the study, the involvement of university investigators and the eligibility testing procedure. Patients indicating a wish to participate will be invited by telephone for eligibility testing. After obtaining a first informed consent, investigators will perform the eligibility testing and, if proven eligible, obtain a second informed consent to participate in the study before proceeding to the baseline assessment.

In the Netherlands, we will include COPD patients from GPs registered in the GP research network of the Department of General Practice, University of Amsterdam (HAG-net Academic Medical Centre) and Zorggroep Almere. The HAG-net Academic Medical Centre consists of 7 health care centres, ± 45 GPs and ± 45,000 patients. The Zorggroep Almere consists of 22 health care centres, ± 120 GPs and ± 165,000 patients. GPs will identify potentially eligible patients through the electronic patient charts and will send them study information and an invitation letter. We will use the 'opt-out method', meaning that patients who are *not *interested to participate or who do *not *permit us to telephone them have to return a reply card indicating so. By telephone, study personnel invites for eligibility testing all patients who did not respond via the reply card within 10 days. After obtaining informed consent to participate in the study, investigators will perform the eligibility testing and, if proven eligible, proceed to the baseline assessment.

### Eligibility testing

The study personnel will assess whether the patients have COPD stage II–IV using portable and hand-held spirometry (EasyOne Model 2001 diagnostic spirometer, ndd Medizinaltechnik, Zürich, Switzerland). This spirometer uses ultrasonic flow measurements and determines several lung function parameters. For our purpose, we will determine postbronchodilator FEV1 and Forced Vital Capacity (FVC) as a measure for airflow obstruction and the Inspiratory Reserve Volume (IRV) as a measure for hyperinflation. Ten minutes before measuring lung function, patients will inhale two puffs of 100 μg salbutamol through a spacer. Informing the patient about the study, obtaining informed consent and lung function testing will require about 30 minutes.

### Baseline assessment

Baseline assessment is standardized and includes history taking, completion of self-administered questionnaires, testing of exercise capacity and blood sampling. To ensure comparability across centres and countries, we have developed and pilot tested case report forms and instructions for testing. Investigators will meet at least twice a year to ensure that the baseline assessment remains identical in both countries. The baseline assessment takes about 60 minutes to complete.

The following data will be ascertained at baseline by study personnel:

#### Patient history

• General information: date of birth, sex, living situation, (former) occupation and education.

• COPD-specific: study personnel will enquire after the year the diagnosis of COPD was made, smoking history, current smoking and smoking exposure at home. The number of pack years (1 pack year = smoking 20 cigarettes per day for one year) will be calculated. We will rely on patient-reported smoking. Ideally, smoking is determined chemically using carboxyhemoglobin (CO-Hb). Although CO-Hb will be measured in this study, it is likely that GPs using the risk index in the future will determine current smoking by interview instead of measuring CO-Hb.

• Exacerbations: since ascertainment of exacerbations is challenging [17,18], we will use several approaches to identify them. First, study personnel will ask the patients how many exacerbations they have had in the previous year. Second, this will be checked in the electronic or hand-written patient charts for documented in- and outpatient treatments. To be counted as an exacerbation, 2 criteria must be fulfilled;

**1**. unscheduled physician contact in a hospital, private practice or by telephone for worsening of dyspnea, cough, increased sputum production and/or a change in sputum color

**2**. electronic or hand-written documentation of a new prescription or a dosage increase of systemic steroids and/or new prescription of an antibiotic.

• Dyspnea scale: we will use the Medical Research Council (MRC) dyspnea scale [19]. The MRC scale ranges from 0 (not troubled by breathlessness except on strenuous exercise) to 4 (too breathless to leave the house or breathless when (un)dressing).

• Chronic cough and phlegm: we will use the questions about cough and phlegm from the Swiss Cohort Study on Air Pollution and Lung Diseases in Adults (SAPALDIA) Questionnaire [20]. In Switzerland, the original version in German will be used; in the Netherlands a Dutch version will be used. Two translators independently translated the German version of the SAPALDIA questionnaire into Dutch. After consensus, another translator, unaware of the original German version, backtranslated this Dutch version of the SAPALDIA questionnaire and the backtranslated version appeared to be similar to the original German version.

• Comorbidities: by asking the patient and screening the patient chart, study personnel will register whether the patient has been diagnosed with cardiovascular disease, cerebrovascular disease, diabetes, current or previous cancer, musculoskeletal disease or other chronic disease. Study personnel will also register the current health status, e.g. current infections.

• Drugs: study personnel will register the prescribed drugs at that moment as documented in the patient chart, including yearly influenza and pneumococcal vaccination.

• Other therapies: study personnel will register if the patients have had long-term oxygen therapy, physical training, lung surgery, lung revalidation etc.

• Body Mass Index: study personnel will measure weight and height to derive the Body Mass Index (BMI).

#### Questionnaires

• COPD-specific health-related quality of life: we will use the widely used and validated Chronic Respiratory Questionnaire (CRQ) in a Dutch and German version [21,22]. The 20 questions of the CRQ provide summary scores for four domains that are deemed important to COPD patients (dyspnea, fatigue, emotional function, and mastery). Patients answer each question on a seven-point scale to express the degree of disability from 1 (maximum impairment) to 7 (no impairment). The total score and score per domain can be calculated by using the mean value ranging from 1 to 7. We will use the self-administered version that takes 5–10 minutes to complete. A change of 0.5 in CRQ domain scores represents the minimal important difference [23].

• Feeling thermometer: the Feeling thermometer is a validated visual analogue scale presented as a thermometer with 100 marked intervals [24-26]. The worst (dead = 0) and the best (perfect health = 100) health states are defined anchors and facilitate comparisons between individuals and groups. We will ask patients to reflect in their score how they have felt in the last seven days.

• Self-efficacy instrument: self-efficacy is the patient's belief in his or her skills to manage the illness. It is associated with medication compliance and might be a prognostic factor. We will use a short COPD-specific instrument to measure the patient's self-efficacy. It contains 3 questions about the management of their illness on a five-point scale from 1 (not confident) to 5 (very confident).

• Anxiety and depression: for affective disorders, common in COPD patients and contributing to health-related quality of life, we will use the Hospital Anxiety and Depression Scale (HADS) [27]. The HADS has been developed and validated to assess symptoms of anxiety and depression in patients with physical impairment. There are seven items for each domain (anxiety and depression) with statements on emotions and emotional situations. Patients express their agreement with the statements on a scale from 0 to 3. Domain scores are calculated by summing up the scores for the seven items resulting in scores from 0 (no depression/anxiety) to 21 (depression/anxiety very likely to be present). Scores ≥ 8 indicate that there is an increased probability of an affective disorder being present. A change of 1.5 in HADS domain scores represents the minimal important difference [23,28]. We will use the self-administered version that takes around 5 minutes to complete.

• Self-reported activity: we will use the Physical Activity Questionnaire (LAPAQ) from the Longitudinal Ageing Study Amsterdam (LASA) [29]. The LAPAQ is a validated questionnaire available in English and Dutch that covers the frequency and duration of walking outside, bicycling, gardening, household activities and sport activities during the previous two weeks. The questionnaire takes 5–10 minutes to complete. Since there was no German version available we have developed one. Three translators independently translated the Dutch LAPAQ into German. After a consensus meeting a first version of the LAPAQ was generated that was considered to be identical to the Dutch version. We then pilot tested the German LAPAQ for its comprehensibility in COPD patients. Another translator unaware of the original Dutch version backtranslated the LAPAQ and the backtranslated version was compared with the original LAPAQ in order to exclude conceptual differences. A formal validation process of the German LAPAQ is currently ongoing.

There is one difference between the Dutch and German LAPAQ. In the Netherlands walking and bicycling for transportation purposes are considered as common daily activities and not as sports activities. This is not the case in Switzerland where bicycling is mostly a sports activity. Therefore, we decided to add a question to the German version containing the frequency and duration of walking uphill because walking uphill is a daily activity for many people in Switzerland. We made this addition in collaboration with the developers of the original LAPAQ.

To counteract potential order effects that might result if patients are, for example, tired after one questionnaire or more tired completing the LAPAQ than completing the CRQ, we will randomize the order of questionnaire administration. Patients are assigned one out of six different orders. We will use a computerized block randomization to generate the randomization list.

#### Exercise Capacity

We will use 2 tests to measure the exercise capacity.

• Sit-to-stand test: this test is performed using a chair without arm rests. The test is first demonstrated by study personnel. Patients are requested to hold their hands on their hips and to complete the sitting and standing positions as correctly and as fully as possible without using the arms for support. After a cue, patients stand upright and without delay sit down again, repeating the procedure as many times as possible in a 1 minute period at a self-selected speed in which they feel safe and comfortable (taking rests at their own discretion). Care is taken to be in full extension motion and approximately 90 grade flexion motion in knee joint. The number of completed repetitions is recorded. The patients are permitted to have rest periods to complete 1 minute [30].

• Handgrip test: we will use the Hand Dynamometer (JA Preston Corp, Ontario, Canada) to assess grip strength of both hands. The measurements are performed while the patients are seated with the shoulders adducted, elbows flexed to 90° and forearms in the neutral position. The test will be performed three times in each hand.

#### Blood sample

Five tubes (two heparin tubes, one serum tube and two EDTA tubes) of venous blood will be taken to test for parameters with potential associations with COPD-specific health-related quality of life decline, exacerbations, death and for future testing of blood markers and genetic factors in order to protect our study from early out-of-datedness. Although genetic testing is not routine yet in COPD, there are a number of polymorphisms with potential prognostic significance. We comply closely with the guidelines for biobanks of the Swiss Academy of Medical Sciences [31]. To ensure correct sampling, storage and genetic analysis, the samples will be coded. The decoding is stored at the Horten Centre in Zurich, Switzerland, and the Academic Medical Centre in Amsterdam, the Netherlands, and is only accessible to the investigators. The blood samples will be taken to the laboratories in Zurich and Amsterdam by the study personnel. One heparin tube will be used for determining carboxyhemoglobin and the second for testing blood markers (C-reactive protein, total cholesterol, high-density lipoprotein cholesterol, low-density lipoprotein cholesterol, triglycerides, creatinin, bilirubin, alanine-aminotransferase, gamma-glutamyltransferase and glucose). The remaining blood and the serum tube will be distributed to four aliquots and stored at -80°C in freezers located at University Hospitals of Zurich and Amsterdam. One EDTA tube will serve to extract DNA and the second to count leucocytes and to perform a differential blood count. The blood samples will be stored for a maximum duration of 20 years.

### Follow-up

Currently, funding is assured for at least two years of follow-up. For an extension of the follow-up, additional funding is required. We will contact patients half-yearly by telephone to ascertain outcomes and to update the patients' profile using the baseline patient history form and we will send them the questionnaires. After two years we will additionally measure lung function and exercise capacity again. We will ask patients about exacerbations during the follow-up interviews. In addition, the Swiss GPs will be asked to complete a form each time an exacerbation occurs and fax it to the study center. The Dutch investigators will review the patient charts for possible exacerbations. Thus, this combination of means to document exacerbations will minimize the chance of missing exacerbations requiring medical treatment, Also, for each patient, an independent and blinded adjudication committee (2 experienced pulmonologists and 1 GP) will check all clinical evidence from all available documents to judge whether the doctor or hospital visits were due to an exacerbation and to distinguish these from other events mimicking exacerbations such as heart failure.

To take into account changes in the patients' profile over time, we will interview patients during follow-up about dyspnea, cough, physical activity, smoking status and treatment as described above. Based on experience in earlier studies, these follow-up interviews will require about 25 minutes.

### Statistical analysis – prediction model

In choosing an approach to the statistical analysis, the use of the risk index in practice should be kept firmly in mind. The goal is prediction of the course of illness as expressed in health-related quality of life, risk of exacerbation and mortality. In principle, the CRQ has four domains (dyspnea, fatigue, emotional function, and mastery) and domain-specific predictions probably will trigger different therapeutic actions. So, potentially, seven different prediction models can be built: 4 on the CRQ domains, one for the overall CRQ score, one for exacerbation risk, and one for mortality, respectively. These models are likely to have at least some predictors in common. We envision that a practitioner will collect all predictors and receive a pattern of 7 predictions to discuss with the patient and base the therapy on. The added value of information whose collection requires more time (sit-to-stand test, questionnaires) will be systematically assessed. Obviously, the different endpoints require the use of different regression techniques: linear regression (CRQ), Cox regression for recurrent events (exacerbations) and Cox or logistic regression for mortality.

In clinical terms we envision the use of a prediction model in the following situations. First, in patients who visit the practice and are first diagnosed as having COPD. And second, in patients known to have COPD, who are identified through the electronic patient charts for a scheduled follow up visit. Our model will not be optimally suitable for use at unscheduled visits, triggered perhaps by exacerbations due to our exclusion criterion on current exacerbations.

Issues important in constructing multivariable models depend on the purpose of the multivariable model (correcting for confounding in studying causal effects, exploratory analyses that try to identify independent risk factors, or prediction models). Even with the purpose in mind, there seems to be consensus that there is no consensus on how one should build multivariable models [32-34]. Here we will restrict to issues important in constructing prediction models. In particular, the issue of variable selection is contentious. Very often, subject matter knowledge is not of much help, since that has already been incorporated into the questionnaire development and the measurement of other (e.g. blood-based) determinants. Such questionnaires most often contain more items than a realistic model may tolerate. We favor parsimonious models because most clinicians have little time. Even data reduction techniques such as principal component analysis applied to many predictors, as proposed by Harrell [33], although facilitating efficient model fitting, still forces physicians to collect a lot of data. Usually there is no time to do that, although (in the future) there may increasingly be exceptions where part of the information may be collected from electronic patient charts or in the waiting room through an internet connection or from home prior to the actual visit. Most experts think that univariable screening of predictors should not be performed and we will avoid that.

Missing values in limited amounts and if scattered throughout different predictors may be dealt with by multiple imputation although it may require additional software programming to perform for example bootstrapped variable selection in more than one imputed data set and elegantly integrate the results from each imputed data set into a single model. By contrast, many missing data on just one or a few tests may indicate that such data will end up missing in practice too, so imputation and incorporation of such a test in a prediction rule seems unwise.

Royston and Sauerbrei emphasize the importance of modeling continuous predictors correctly, avoiding unnecessary categorization They argue that multivariable fractional polynomials may be seen as a flexible and convenient compromise between ultra-flexible but potentially unstable local influence models, such as (smoothing) splines and relatively inflexible global influence models, such as for example conventional polynomials [34]. We think that they argue their case convincingly and will explore multivariable fractional polynomials for our continuous predictor candidates such as age, exercise capacity test results and blood values.

The biggest issue of contention seems to be the one of variable selection. There is the choice between full models (which, as stated above, we think are impractical due to physicians' time restraints), backward elimination, shrinkage, lasso (least absolute shrinkage and selection operator) or least angle regression (LAR) and finally, one we think has much potential, the garotte [34-36]. The garotte is a shrinkage approach in which the degree of shrinkage is reduced as the predictor's regression coefficient gets larger. This is attractive since, according to Copas, large coefficients do not need (much) shrinkage. Lasso and general shrinkage may shrink large coefficients too much. A drawback of the garotte is that it starts with a full model which may be too large to fit. We expect to be able to explore the garotte if we succeed in programming it. The final model may still be subjected to enhanced bootstrap procedure to estimate over-optimism. This is usually expressed as the difference between areas under the curve or Brier scores. We will explore any over-optimism in the predicted values and their corresponding confidence intervals.

In much of the literature on prediction of cardiovascular disease, ongoing treatments in observational cohorts are not (explicitly) accounted for. This seems to be incorrect because it is easy to envision that the prognosis of a patient with high blood pressure while on antihypertensive treatment differ from a patient with high blood pressure without any treatment. For example in COPD, the prognosis of a patient with a low MRC score while on bronchodilators can differ from the prognosis of a patient with the same MRC score without bronchodilators. In ICECOLD ERIC we collect data on treatment (changes) and intend to use them in the models where necessary.

We will have several options to validate our prediction model. For example, the Dutch cohort could be the derivation set and the Swiss cohort the validation set or vice versa. One of other possibilities is that half of each cohort could be used as derivation set and the other half as validation set. Or, we can even combine all data to derive the model and test the model via bootstrap. At the time of writing, we have not decided which route we will follow. At the time we need to make that decision, we will try to find the best solution at that moment according to the statistical literature.

Discrimination of the model(s) will be visualized in high resolution histograms and summarized as 5^th^, 10^th^, 25^th ^50^th^, 75^th^, 90^th^, and 95^th ^centiles of these, Brier score and the area under the receiver operating characteristics curve (ROC) with 95% confidence intervals (overall discrimination)[37]. Using the regression coefficients of the independent diagnostic indicators, an easy to use, multivariable diagnostic rule (clinical index) will be derived, consisting of relevant tests and their diagnostic values [38]. Calibration will be assessed using Hosmer Lemeshow tests and visualized using calibration plots (observed versus expected (or rather, model-based) probabilities).

### Statistical analysis – selecting patient management

A prediction rule is not a decision rule [39]. For physicians, it may be unclear how predictive information may be employed to change a patient's management beneficially. We would like to incorporate knowledge on treatment effects into our model(s) to guide physicians and patients in their decisions on how management should change given the predicted probabilities that a predictive model provides. In that respect, separate prediction of long-term outcomes for the sub domains of the CRQ may be much more unequivocal than a prediction of the total score in one or two years time. So, it is still attractive to explicitly model effects of treatment as to provide physician and patient with predictions conditional on particular treatments. Although the ICECOLD ERIC cohort(s) contain some information on treatment effects, we expect that the combined evidence from RCTs on COPD or good meta-analyses thereof may be a more fruitful source to see how predictions may be altered beneficially. This topic is currently being debated by our group and we cannot be more specific here.

### Sample size

The number of covariables that we will consider for the regression analysis is roughly 20, and using the rule that at least ten times as many patients are needed than covariables considered, we will need at least 200 patients per population.

### Data collection and quality control

We will implement a series of measures to ensure high data quality.

1. Site investigators will collect the data using standardized forms.

2. All data will be collected and entered into a single database in MS-Access at the Academic Medical Centre provided by the Department of Clinical Epidemiology, Biostatistics and Bio-informatics. All data will be double entered and checked by a compare procedure.

3. International investigator meetings will be held (at least) twice yearly for all staff involved in data collection to detect any divergence in data collection procedures. This involves site mutual visits whenever possible.

4. Local investigators meetings will be held monthly, or more frequently if needed, to discuss recruitment of patients and problems in conducting the study.

5. E-mail will serve as the first line of non-urgent communication between research team members.

6. In Switzerland, monthly reports will be prepared by the principal investigator and sent to all GPs to inform about the number of patients recruited and important issues concerning the conduction of the study.

## Discussion

Management of COPD is challenging due to the various treatment options and the heterogeneous character of the disease. The actual (as opposed to ideal) assessment and treatment choices today are quite similar for all COPD patients and are often based on too few disease characteristics. This often leads to undertreatment of patients with severe COPD and overtreatment of patients with mild COPD. An example of overtreatment is the widely prescribed inhaled corticosteroids in mild patients. Results from recent studies suggest that GPs may not have methods available to sufficiently consider severity of disease for treatment decisions [3-11].

In preventive cardiovascular care, treatment decisions are based on the cardiovascular risk profile of the individual patient in primary care. Based on the most important predictors, patients fall into a specific risk profile. These profiles are formally coupled with treatment effects and costs considerations. Based on these components, treatment recommendations may be made. We believe that this so called risk index-guided treatment for specific patient profiles may well be applicable to COPD too.

The aim of this study to develop and validate a practical COPD disease risk index for the primary care will individualise and improve COPD management. Assessment of disease severity should not focus solely on lung function. An increasing body of evidence shows that additional information should be ascertained that better reflects disease severity than lung function [2,12-15].

We expect that after this study, the disease risk index will be ready for use in primary care. It will divide COPD-patients into specific risk profiles (low, medium and high risk) for each of the outcomes; COPD-specific health-related quality of life (domain-specific), exacerbation risk and mortality. In a second step we will incorporate treatment advices into the model by using data mainly from pertinent RCTs and meta-analyses. Ways to incorporate treatment advices into the prediction model may be challenging and are currently being explored by our team. In the development process of the cardiovascular risk model, the relative risk (RR) was treated as constant across all risk profiles.

Thus a predicted risk of cardiovascular death within 10 years of 30% may be reduced by RR = 0.8 to 24% (absolute risk reduction = 6%) whereas the same RR reduces a 3% predicted risk to 2.4% (absolute risk reduction = 0.6%). Adding costs considerations may lead to a recommendation to treat the former, and not the latter patient. It is unclear whether in our study, examining COPD, we will have the luxury of finding convincing evidence of constancy of relative risks (or odds ratios) of different treatments. If not, we will have to develop more refined methods.

Using the risk score that we will develop, the management of COPD in primary care will improve. Treatment decisions will be tailored better to the needs of the individual patient, resulting in less unnecessary treatment prescriptions, less exacerbations and a better COPD-specific health-related quality of life. We also expect that COPD management will become more cost-effective.

## Competing interests

The authors declare that they have no competing interests.

## Authors' contributions

LS, GR and MP drafted and revised the manuscript. All authors participated in development of research protocols and in the design of the study. All authors read and corrected draft versions of the manuscript and approved the final manuscript.

## Pre-publication history

The pre-publication history for this paper can be accessed here:


